# Developing momentum in vanishing index photonics

**DOI:** 10.1038/s41377-022-00846-0

**Published:** 2022-05-20

**Authors:** Nathaniel Kinsey

**Affiliations:** Department of Electrical & Computer Engineering, Virginia Commonwealth University, Richmond, VA 23284 USA

**Keywords:** Optical materials and structures, Optical physics

## Abstract

Refractive index invariably describes the speed at which light passes through materials, and subsequently its perceived momentum. But what happens to these quantities as the index becomes zero? A new work explores this question, highlighting how momentum in near-zero-index materials affects linear optical processes.

The first day of an introductory optics course likely includes a discussion of waves and the concept of the refractive index, the ability of a material to alter the speed at which light moves, and how this fact can be used to design any number of useful optical elements. As is clear, the refractive index of a material is fundamental to our ability to manipulate light and design its amplitude, phase, and relative dispersion^1,2^.

In fact, many optical effects (reflection/refraction, resonance, confinement, etc.) and the performance of subsequent devices can be linked to the index contrast available in a given implementation. Thus, a key focus of optical materials research has centered on expanding our understanding of the refractive index^2–4^ and pushing its boundaries. From identifying fundamental limits^5,6^, going to the extremes of index^7,8^ as well as engineering metamaterials^9–13^, our ability to tailor the index has grown significantly, having led to a host of exciting new developments as the refractive index is taken to its ‘extremes’^14–19^.

In recent years, the study of near-zero-index (NZI) materials has arisen^20–22^ and is closely linked to the concept of materials with other vanishing properties such as epsilon-near-zero (ENZ), mu-near-zero (MNZ), and epsilon-mu-near-zero (EMNZ). Such materials constitute a ‘lower-extreme’ to the index and are commonly realized with metallic films or heavily doped semiconductors, phononic materials, and through effective structures which blend materials or modify the index of a specific mode^23^. In particular, NZI and ENZ have gained interest due to their relative ease to implement in a wide variety of applications, large index contrast that can be realized with traditional dielectric and semiconductor materials, as well as the host of unique optical effects that occur such as super coupling^24^, emission tailoring^25^, long-range interactions^26^, and geometry invariant photonics^27^.

While the frontier of utility has grown rapidly within the metamaterials and plasmonics communities^28–30^, a theoretical understanding of the interactions of light inside such materials has generally lagged experimental demonstrations. For example, despite experimental works in nonlinear interactions being realized in 2015 by several groups^31,32^, a robust theory was not established until 2020^33^.

In the work by Lobet et al.^34^, the authors seek to fill one such gap by expanding upon the fundamental understanding of momentum in NZI materials and its sub-classes (ENZ, MNZ, and EMNZ). In particular, the authors place the Minkowski and Abraham momentum descriptions into the context of vanishing property materials, illustrating their connection to the phase and group velocity of light, respectively. Situations such as spontaneous emission, lasing, and microscopy are presented where the materials are illustrated to eliminate slit interference, ‘hide’ objects by filtering k-vectors, and facilitate spatial translations of fields. While the quantities described herein will be readily familiar to the general optics researcher, the discussion centered around momentum provides a different, yet straightforward, angle through which one may view such interactions and evaluate materials that is also tractable to readers outside of the NZI-community.

While the implications in linear optics are one avenue, as discussed within the paper, the framework also has connection with the growing area of nonlinear optical interactions in vanishing property materials as well^35^. In particular, the area of space-time nonlinear interactions, where strong spatial (Δn ~ 1) and temporal (Δt ~ 300 fs) changes in the refractive index are driving interesting works in optical switching^32,36^, frequency conversion^37–40^, and time-refraction^41,42^. In such interactions, wave-like and particle-like interactions can coexist, leading to time-varying reflection coefficients and phase accumulation as well as Doppler-like frequency shifting and momentum exchange. While works are beginning to highlight the roles of certain components^40,43^, there is still an opportunity to solidify the theoretical foundation. These nonlinear responses represent an intriguing regime to employ the momentum framework and observe the relative roles of each momentum description. Furthermore, the resulting descriptions also represent an interesting angle to view light-matter-interactions in more complex devices which combine nanostructures with vanishing property materials to tailor the effective index and are likely to find application in these emerging areas as well.
